# Microbiome analysis of Brazilian women cervix reveals specific bacterial abundance correlation to RIG-like receptor gene expression

**DOI:** 10.3389/fimmu.2023.1147950

**Published:** 2023-04-25

**Authors:** Alan Messala A. Britto, Juliana D. Siqueira, Gislaine Curty, Livia R. Goes, Cintia Policarpo, Angela R. Meyrelles, Yara Furtado, Gutemberg Almeida, Ana Lucia M. Giannini, Elizabeth S. Machado, Marcelo A. Soares

**Affiliations:** ^1^ Departamento de Enfermagem Materno-Infantil (DEMI), Faculdade de Enfermagem (FEnf), Universidade do Estado do Rio de Janeiro (UERJ), Rio de Janeiro, Brazil; ^2^ Programa de Oncovirologia, Instituto Nacional de Câncer (INCA), Rio de Janeiro, Brazil; ^3^ Programa de Pós-Graduação em Infecção HIV/aids e Hepatites Virais, Hospital Universitário Gaffrée e Guinle (HUGG/Ebserh), Universidade Federal do Estado do Rio de Janeiro (UNIRIO), Rio de Janeiro, Brazil; ^4^ Laboratório de Genômica Funcional e Transdução de Sinal, Departamento de Genética, Instituto de Biologia, Universidade Federal do Rio de Janeiro (UFRJ), Rio de Janeiro, Brazil; ^5^ Instituto de Ginecologia, Universidade Federal do Rio de Janeiro, Rio de Janeiro, Brazil; ^6^ Escola de Medicina e Cirurgia da Universidade Federal do Estado do Rio de Janeiro, Universidade Federal do Rio de Janeiro (UNIRIO), Rio de Janeiro, Brazil; ^7^ Hospital Universitário Clementino Fraga Filho, Universidade Federal do Rio de Janeiro, Rio de Janeiro, Brazil

**Keywords:** microbiome and dysbiosis, innate immunity recognition, virome analysis, human papilloma virus (HPV), squamous intraepithelial lesion of the cervix, RIG-I-like receptor family, toll-like receptors (TLR), anellovirus

## Abstract

The relationship among microbiome, immunity and cervical cancer has been targeted by several studies, yet many questions remain unanswered. We characterized herein the virome and bacteriome from cervical samples and correlated these findings with innate immunity gene expression in a Brazilian convenience sample of HPV-infected (HPV+) and uninfected (HPV-) women. For this purpose, innate immune gene expression data were correlated to metagenomic information. Correlation analysis showed that interferon (IFN) is able to differentially modulate pattern recognition receptors (PRRs) expression based on HPV status. Virome analysis indicated that HPV infection correlates to the presence of *Anellovirus* (AV) and seven complete HPV genomes were assembled. Bacteriome results unveiled that vaginal community state types (CST) distribution was independent of HPV or AV status, although bacterial phyla distribution differed between groups. Furthermore, TLR3 and IFNαR2 levels were higher in the *Lactobacillus* no iners-dominated mucosa and we detected correlations among RIG-like receptors (RLR) associated genes and abundance of specific anaerobic bacteria. Collectively, our data show an intriguing connection between HPV and AV infections that could foster cervical cancer development. Besides that, TLR3 and IFNαR2 seem to create a protective milieu in healthy cervical mucosa (*L.* no iners-dominated), and RLRs, known to recognize viral RNA, were correlated to anaerobic bacteria suggesting that they might be related to dysbiosis.

## 1 Introduction

Cervical cancer is the fourth most prevalent type of female tumor worldwide and the third most common cancer among Brazilian women (1, 2). Human papillomavirus (HPV) infection is necessary for cervical cancer development but other factors such as viral persistence, immunodeficiency and smoking are also associated with the disease (1, 3, 4). Moreover, cervical mucosa immune regulation and local microbiota also play pivotal roles in this process (5).

The female reproductive tract (FRT) has several protective components, including antimicrobial peptides (AMPs – small proteins released by different cell types with antimicrobial activity), and immune and epithelial cells (6). Besides acting as a physical barrier, epithelial cells express a wide repertory of pattern recognition receptors (PRRs), such as Toll-like receptors (TLRs) and RIG-like receptors (RLRs), responsible for recognizing conserved pathogen-associated molecular patterns (PAMPs) like nucleic acids and lipopolysaccharides from bacteria, fungi or viruses. TLRs are transmembrane receptors found in cell surface and intracellular membranes, that recognize LPS (TLR4) or nucleic acids (TLR3, TLR7, TLR8 and TLR9), while three receptors comprise the cytoplasmic RLR family – RIG-I, MDA5 and LGP2 – responsible for recognizing nucleic acids, especially non-self RNA forms (7). The pathways activated by these receptors are regulated at several levels, for instance, RIG-I activity is positively regulated by TRIM25 (8) while RNF125 mediated ubiquitination of both RIG-I and MDA5 results in their degradation (9). Once activated, both TLRs and RLRs, *via* different adaptor proteins, lead to engagement of transcription factors such as NFκB, IRF3, IRF7 that regulate cytokine and IFN production (6, 10). Pathogens impair these innate responses in several ways, including by handling ubiquitination machinery. In high-risk HPV (hr-HPV) infection, *e.g*, UCHL1 de-ubiquitinates TRAF3 and NEMO (adaptor proteins of TLRs and RLRs pathway), impairing cytokines and IFN-I production (11). These genes and cytokines are differentially expressed when comparing cervical cells from HPV-infected (HPV+) and uninfected (HPV-) women and may have important roles in HPV persistence or clearance and in susceptibility to STIs such as HIV infection (12–15). Furthermore, several studies suggest important roles of PRR expression and host microbiota in health and disease at mucosal sites (16–18), but little is known about the FRT.

Women’s vaginal bacteria are grouped based on community composition into five “community state types” (CSTs): CST I, II, III, IV and V, dominated by *Lactobacillus crispatus, L. gasseri, L. iners*, anaerobic bacteria (*Gardnerella, Megasphera, Sneathia and Prevotella*) and *L. jensenii*, respectively (3, 19). A healthy vaginal environment is dominated by *Lactobacillus* and shows low bacterial diversity (CSTs I, II or V), while a decreased concentration of *Lactobacillus* other than *L. iners*, increased diversity and anaerobic dominance typify a transitional mucosa (CST III-dominated) or dysbiosis (CST IV-dominated), the latter also known as bacterial vaginosis (BV) state (3, 15, 20). BV is commonly found in women of black (African) and Hispanic ethnicities and is also influenced by smoking and sexual activity (19, 21–24). Several studies associate CST III or IV and increasing microbiome diversity with HPV infection, cervical intraepithelial neoplasia (CIN), invasive cervical cancer (3, 25, 26) and HIV infection (27).

An initial characterization of the vaginal bacteriome of Brazilian women indicates that women under reproductive age analyzed in the study grouped into the five CSTs. Nevertheless, a predominance of CST III (36.5%), CST I (30.5%) and CST IV (27.4%) was found. In this population, factors like smoking, number of partners, demographic region of origin and vaginal douching were risk factors for dysbiosis, while ethnicity, educational level and condom use had no impact on CST dominance (28). Besides bacteria, the vaginal microbiota is also composed of a viral community able to infect either eukaryotic or prokaryotic cells. Several studies suggest that the host virome has an important role in innate and adaptative mucosal immunity and is linked to the development of BV, infertility, and adverse pregnancy outcomes such as prematurity and intrauterine growth restriction (29–31). Despite its importance, vaginal virome data are still scarce and further studies are required as they might contribute to the understanding of this complex system and how its components relate to each other in health and disease. To contribute to this understanding, herein we characterized the virome and bacteriome from cervical samples and correlated these findings with innate immunity gene expression. Although our sample size is limited, we obtained significant correlations when comparing HPV-infected or uninfected women. Virome data indicated that HPV infection is correlated to the presence of *Anellovirus*. Also, bacteriome results suggested that phyla distribution is different depending on HPV and *Anellovirus* status. When gene expression was assessed, we observed that type I IFN expression is correlated to TLR9 expression in HPV+ women, while it is correlated to TLR4 expression in HPV- samples. Moreover, when bacteriome data were assessed in conjunction with gene expression data, TLR3 and IFNαR2 levels were higher in the *Lactobacillus* no iners-dominated mucosa and we also detected correlations among RIG-I, MDA5, TRIM25, RNF125 and abundance of specific anaerobic bacteria.

## 2 Material and methods

### 2.1 Study patients, sample collection and ethical statements

Study participants were enrolled at Instituto de Ginecologia (IG), Universidade Federal do Rio de Janeiro (UFRJ), Brazil. This was a convenience sampling from studies of characterization of sexually transmitted infections (32) and cervical immunity analysis (12). During medical consultation in the cervical pathology and colposcopy outpatient clinic or in the gynecology ambulatory, we collected cervical epithelial cells with one cell scraper and two endocervical cytobrushes; 4 ml of whole blood was also collected from each woman. All material was used to diagnose STIs and perform other experiments as previously described (12, 32). In this study, we included cervical cells of a total of 22 women above 18 years of age who tested negative for chlamydia, trichomoniasis, syphilis, gonorrhea, HIV and HBV, and that had their innate immune genes previously quantified. They were classified as healthy women (HPV- n = 11; HPV-negative without cervical lesions) or HPV+ (n = 11; high-risk HPV-positive with high-grade squamous intraepithelial lesions (HSIL)) based on Sanger sequencing (12). Briefly, nested polymerase chain reaction (PCR) of gDNA was performed (My9/My11 primers followed by GP5+/GP6+ primers) and PCR product purified and sequenced by Sanger method. Sequences were compared to HPV sequences available in the GeneBank database (NCBI, NIH, Bethesda, USA) and the HPV type was assigned. The study was approved by the Ethical Committee of Instituto de Puericultura e Pediatria Martagão Gesteira, UFRJ (approval number: 49035215.4.0000.5264).

### 2.2 Circular DNA enrichment, sequencing and virome analysis

Total genomic DNA (gDNA) was extracted from cervical epithelial cells with the HiYield™ Genomic DNA Mini Kit (Blood/Bacteria/Cultured Cells) (Real Genomics, Taiwan). Circular DNA was enriched by rolling circle amplification (RCA) with the Illustra TempliPhi Amplification kit (GE Healthcare Life Sciences, Piscataway, NJ, USA). We prepared sequencing libraries using two nanograms of purified RCA product and the Nextera XT DNA Sample Preparation kit (Illumina Inc., San Diego, CA, USA). One HPV- patient was excluded from downstream analyses due to lack of sufficient gDNA. Samples were sequenced in an Illumina MiSeq platform (2 x 100 nt reads).

Reads with Phred quality score below 28 were trimmed with Sickle (33) and the remaining was mapped to the human reference genome hg19 using BWA (34) to remove human reads. Non-human reads were compared to viral sequences using BLASTX (35) and the virus protein database from RefSeq. Hits with an e-value below 0.01 were submitted to the BLASTX against the GenBank’s non-redundant database. Reads were classified as virus/non-virus and the virus family was defined according to the most similar sequence found in the database. *Retroviridae* virus family was excluded to avoid biased analysis since most reads from this family were removed during the disposal of human reads. Virus relative abundance was calculated by dividing the number of reads assigned to each virus family per the total of trimmed reads submitted to BLASTX. Reads were also assembled to HPV reference genomes obtained from PAVE database (http://pave.niaid.nih.gov) using Geneious R11 (Biomatters, Auckland, New Zealand) and the consensus sequences representing the HPV complete genomes were extracted.

### 2.3 Bacterial community state type characterization

Total gDNA from cervical cells of 21 patients (10 HPV- and 11 HPV+) were used to PCR-amplify the 16S rRNA gene variable V3-V4 regions, generating an amplicon of ~460 bp, with the 16S Metagenomic Sequencing Library Preparation kit (Illumina, Inc.). PCR reactions were carried out using 1X Taq PCR buffer (minus Mg), 1.5 mM MgCl_2_, 0.2 mM dNTP mix, 0.2 µM each primer, 1.5 U Taq Platinum polymerase (Invitrogen, Carlsbad, CA, USA), ~12.5ng of template and final volume was adjusted with RNase/DNase free water (Life Technologies, Carlsbad, CA). The cycling conditions carried out were recommended by the manufacturer of the 16S library. PCR products were visualized by electrophoresis in 1.5% agarose gels and bands of the expected size were purified with the Illustra GFX PCR DNA and Gel Band Purification Kit (Merck KGaA, Darmstadt, Germany). Five microliters of purified 16S rRNA PCR products were then used along with Nextera XT DNA Sample Preparation kit (Illumina, Inc.) to prepare sequencing libraries with distinct barcodes each one. Libraries were diluted to a final concentration of 10 pM and samples were sequenced in an Illumina MiSeq platform (2 x 100 nt reads).

The BCL2FastQ2 Conversion Software (version 2.18, Illumina, Inc.) was used to demultiplex data and convert BCL files to FASTQ file formats. Reads were subjected to FastQC (Babraham Bioinformatics, Cambridge, CBE) for quality analysis, were denoised, filtered and joined using the Divisive Amplicon Denoising Algorithm DADA2 (36) using QIIME2 (37). Taxonomic classification was performed using the q2-feature-classifier QIIME2 plugin and the Greengenes Database (38), and bacterial taxonomic were defined by amplicon sequence variants (ASV) analysis in QIIME2.

Unsupervised hierarchical clustering based on Bray-Curtis dissimilarity and average linkage was applied to define clusters according to abundance and taxa diversity of each sample. The clusters found were associated with vaginal microbiome CSTs classified in previous studies (19). Clusterization, diversity analyses and plots were carried out using the R environment.

### 2.4 Gene expression

Gene expression analyses were carried out as previously described (12). Briefly, total mRNA was extracted from cervical cells and used as template for cDNA synthesis. We prepared customized Taqman array plates, except for IFNγ which we designed primers and used SYBR Green to measure gene expression levels. Reactions were performed in 7500 Fast Real-Time PCR System (Applied Biosystems). Here we selected the following genes to study: RNF125, UCHL1 (seven HPV+ and seven HPV- patients were assessed); TLR3, TLR4, TLR7, TLR9, DDX58 (RIG-I), IFIH1 (MDA5), IFNA2, IFNB1, IFNAR2, TRIM25, (11 HPV+ and 11 HPV-); IFNG (10 HPV+ and eight HPV- patients). TLR3, TLR4, TL7 and TLR9 are receptors of TLR family; DDX58 (RIG-I), IFIH1 (MDA5) belong to RLR family and are regulated by TRIM25 and RNF125; UCHL1 negatively regulate TLR and RLR pathways; IFNα2, IFNβ1 are IFN-I that interact with IFNαR2 amplifying innate immune response; and IFNγ is the type II IFN (IFN-II) mainly produced in adaptative immune response. GAPDH was used as housekeeping gene and relative expression calculated using the 2^-ΔCt^ formula.

### 2.5 Statistical analysis

Sociodemographic data have been summarized with absolute and relative frequency for categorical variables and with mean and standard deviation (SD) or median and interquartile range for numerical variables. Differences between groups were calculated in IBM SPSS Statistics for Windows, version 25.0 (IBM Corporation, Armonk, NY). For categorical variables we used chi-square or Fisher exact test; for numerical variables we used Mann-Whitney U test to compare two groups and Kruskal-Wallis test, with Dunn’s multiple comparisons as a *post-hoc* test to compare three groups. Odds ratio (OR) was calculated using the free online statistical software MedCalc [https://www.medcalc.org/calc/odds_ratio.php]. Correlation analyses were carried out by Spearman’s correlation in SPSS (IBM Corporation), where r corresponds to correlation coefficient. Degrees of correlation were adapted from Schober and colleagues (39) as follow: 0.7 ≤ r ≤1 – strong correlation; 0.4≤ r < 0.7 – moderate correlation; and r < 0.4 – weak correlation.

To compare gene expression levels according to anellovirus status we used Mann-Whitney U test in SPSS (IBM Corporation), and according to CSTs we used Kruskall-Wallis test in GraphPad Prim 9 (GraphPad, California, USA).

## 3 Results

### 3.1 Patient sociodemographic characteristics

Patients were grouped based on their HPV status into HPV- and HPV+ women. HPV+ women were older, but in both groups the number of married, high school educated and smokers or past smokers were similar (**Table 1**). Approximately a quarter of the women used condom and/or hormonal contraceptive methods, and although data did not reach statistical significance, HPV+ women had their first sexual intercourse at a younger age, met more partners in life and had a history of previous STIs (**Table 1**). In order to evaluate if demographic characteristics could affect HPV outcome, we performed Odds Ratio (OR) analyses. Previous STIs were considered a risk factor (OR = 7.875, 95% Confidence interval (CI) = 1.105 – 56.123) and age ≥ 41 yr a protective factor (OR = 0.127, 95% CI = 0.018 – 0.905) for HPV infection.

**Table 1:** Sociodemographic characterization of studied patients.

Characteristics (n)	Overall (22)	HPV+ (11)	HPV- (11)	*p-value*
**Age, mean (SD)**	41.7 (12.8)	35.6 (9.4)	47.9 (13.0)	0.018*
**Married (%)**	13 (59.1)	7 (63.6)	6 (54.5)	1.0
**High school education or more (%)**	13 (59.1)	7 (63.6)	6 (54.5)	1.0
**Actual or past smoker (%)**	10 (45.5)	5 (45.5)	5 (45.5)	1.0
**Tobacco load^a^ (SD)**	23.0 (35.3)	17.3 (27.6)	28.7 (44.3)	1.0
**Previous STI^b^ (%)**	9 (40.9)	7 (63.6)	2 (18.2)	0.08
**Condom use (%)**	4 (18.2)	3 (27.3)	1 (9.1)	0.586
**Hormonal contraceptive use (%)**	5 (22.7)	3 (27.3)	2 (18.1)	1.0
**Age of first sexual intercourse, mean (SD)**	16.3 (3.4)	14.8 (1.9)	17.7 (4.0)	0.091
**No. lifetime partners, mean (SD)**	5.1 (6.9)	7.2 (9.2)	2.9 (2.1)	0.161

a = multiply the number of cigarettes smoked per day, divided by 20 and multiply by the number of years the person smoked; b = Previous HPV or syphilis; n = absolute number; SD, standard deviation; Statistical analysis – Mann-Whitney U Test for numerical variables and Exact Fisher test for categorical variables; * = p < 0.05.

### 3.2 HPV impacts IFN-I inducible gene expression

We evaluated the association between expression of studied genes and HPV status, classifying expression levels in high or low based on the median expression of each gene (low ≤ median; high > median). We observed associations between HPV+ status with IFNα2, TLR7, TLR3 and MDA5 expression using Fisher Exact Test and decided to perform OR analyses. These assessments indicate that HPV infection is linked to high IFN-I expression (IFNα2: OR = 38.3, 95% CI = 1.8 – 820.2; IFNβ1: OR = 12.0, 95% CI = 1.1 – 128.8) and to low levels of PRRs that recognize RNA (MDA5: OR = 0.0368, 95% CI = 0.002 – 0.777; TLR3: OR = 0.022, 95% CI = 0.002 – 0.289; TLR7: OR = 0.026, 95% CI = 0.0012 – 0.558) (**Supplementary Table 1**).

Besides OR evaluation, the correlations among gene expression levels were also calculated using Spearman’s correlation and only the significant results are presented here (**Table 2**). We observed that independently of HPV, IFN-α2, IFN-β1 (type I IFN) and IFN-γ (type II IFN) genes were strongly and positively correlated with each other, as expected (40) (**Table 2**). We then assessed genes that correlated with IFNs, since these cytokines activate several transcription factors that then activate IFN responsive genes, changing expression profiles. We found that genes that correlated with IFNs are different when comparing HPV- and HPV+ samples, suggesting that these cytokines differentially regulate gene expression in the two groups. In HPV+, type I IFNs (IFNα2 and IFNβ1) were positively correlated to TLR9 and negatively correlated to TLR3. In HPV- cervices however, mRNA expression of these cytokines was positively correlated to TLR4 expression, and IFNβ1 mRNA levels were also correlated to MDA5 expression. Interestingly, IFNγ expression was positively correlated to those of MDA5 and RNF125 only in HPV- samples (**Table 2**). The opposite correlation between TLR9 and TLR4 in the two groups corroborates the idea that several genes involved in innate immunity are differentially modulated during HPV infection. We also looked for correlations restricted to each group and found that the expression of MDA5 and RNF125 are correlated only in HPV- women, while expression of RIG-I and TLR3 are correlated solely in the HPV+ group.

**Table 2:** Correlation analysis of gene expression based on HPV status.

HPV-			HPV+		
Parameters	r	*p* value	Parameters	r	*p* value
IFNα2 x IFNβ1	0.955	<0.001	IFNα2 x IFNβ1	0.927	<0.001
IFNα2 x IFNγ	0.891	0.001	IFNα2 x IFNγ	0.927	<0.001
IFNα2 x TLR4	0.700	0.016	IFNα2 x TLR3	-0.674	0.023
IFNβ1 x IFNγ	0.903	<0.001	IFNα2 x TLR9	0.636	0.035
IFNβ1 x TLR4	0.627	0.039	IFNβ1 x IFNγ	0.867	0.001
IFNβ1 X MDA5	0.688	0.019	IFNβ1 x TLR3	-0.674	0.023
IFNγ x RNF125	0.829	0.042	IFNβ1 x TLR9	0.682	0.021
IFNγ x MDA5	0.632	0.05	RIG-I x IFNαR2	0.645	0.032
MDA5 x RNF125	0.821	0.023	RIG-I x TLR4	0.755	0.007
TLR4 x TLR9	-0.609	0.047	RIG-I x TLR9	0.618	0.043
TLR9 x TRIM25	0.618	0.043	RIG-I x TRIM25	0.936	<0.001
			TLR4 x TLR9	0.836	0.001
			TLR4 x TNFα	0.648	0.043
			IFNαR2 x TRIM25	0.664	0.026
			IFNαR2 x TLR3	0.674	0.023

r = correlation coefficient; Degree of correlation = strong correlation: 0.7 ≤ r ≤1 or -1 ≤ r ≤ -0.7; moderate correlation: 0.4 ≤ r < 0.7 or -0.7 ≤ r < -0.4; weak correlation: 0 < r < 0.4 or 0.4 < r <0; negative correlation: r < 0.

### 3.3 Virome analyses

We enriched circular DNA using RCA to characterize circular DNA viruses in cervical samples. The total number of reads obtained per sample after trimming out low-quality reads was 21,535,051 (average: 1,025,479 ± 418,145.8). Overall, we detected two viral families, *Papillomaviridae* (98.8% of viral reads) and *Anelloviridae* (1.2% of viral reads) (**Table 3**). By next-generation sequencing (NGS), we detected HPV reads in a total of nine women, confirming HPV classification based on Sanger sequencing, except for two patients who had no HPV reads (IG13 and IG33), but whose HPV status was confirmed by PCR. Interestingly, patient IG31 was infected by HPV51 with a deletion of 30 nucleotides in the C-terminal DNA binding domain of E2. Reads from five samples assembled seven complete HPV genomes that were classified into HPV types, lineages and sublineages (**Table 3**). Three women were infected by more than one HPV type (IG36, IG62 and IG63), and one was infected by eight different HPVs, most of them hr-HPV types (**Table 3**).

**Table 3:** Patient viral profiles based on Sanger sequencing and next-generation sequencing.

Sample ID	HPV type (Sanger)	AV*	HPV type (no. Reads) - NGS**	Full Genome (Sublineage)
IG03	HPV16	259	HPV16 (278)	HPV16 (D3)
IG13	HPV66	-	-	-
IG19	HPV16	6	HPV16 (30)	–
IG21	HPV31	-	HPV31 (2)	-
IG22	HPV16	–	HPV16 (388)	HPV16 (A1)
IG31	HPV51	4	HPV51 (541)	HPV51 (A1 – del 30nt)
IG32	HPV16	14	HPV16 (1636)	HPV16 (A1)
IG33	HPV31	-	-	-
IG34^a^	–	3	–	–
IG36	HPV66	3	HPV69 (27360); HPV51 (1599); HPV82 (456); HPV52 (153); HPV66 (83); HPV32 (80); HPV89 (19); HPV45 (2)	HPV69 (A4); HPV51 (A1); HPV82 (C2)
IG62	HPV53	-	HPV53 (50); HPV16 (8)	-
IG63	HPV16	21	HPV16 (30); HPV31 (2)	-

(a), HPV negative sample by Sanger sequencing and NGS; *AV, annelovirus read number based on blastx against full genome in Genbank; ** NGS, as determined with Geneious.

Anellovirus (AV) reads were detected in seven women (six out of seven are HPV+) and we decided to group study participants by status of this viral family (AV- = 14 women; AV+ = 7 women) and performed OR analyses. First, we observed that being infected by HPV favors *Anellovirus* infection (*p* = 0.043; OR = 10.8, 95% CI = 1.0 – 117.0). In addition, AV+ women had more partners in life (AV- (median/IQR) = 2.9/2; AV+ = 10/5; *p* = 0.028) and presented more HPV reads in NGS analysis (AV- (median/IQR) = 12/0; AV+ = 2638/152; *p* = 0.002). Nevertheless, no differences in gene expression were detected between AV- and AV+ women (**Supplementary Figure 1**). We also correlated *Papillomaviridae* and *Anelloviridae* read abundance with gene expression levels and observed a negative correlation between HPV abundance and UCHL1 (r = 0.829, *p* = 0.042).

### 3.4 Bacteriome analyses

**Figure 1:**
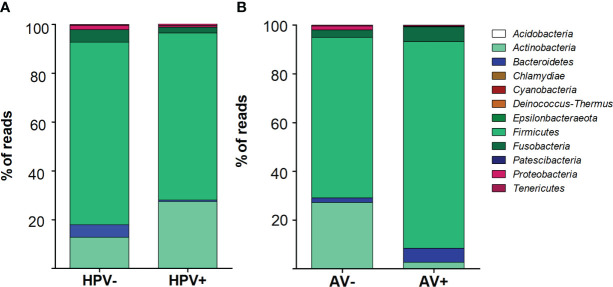
Bacterial phyla distribution depending on HPV **(A)** or Anellovirus **(B)** status. Stacked vertical bar graphs showing the percentage of total reads found in **(A)** HPV- and HPV+ women and **(B)** Anellovirus negative (AV-) and Anellovirus+ (AV+) women. The frequencies of phyla were assessed by chi-square test and were different between groups (p < 0.001).

Total DNA of cervical cells was used as a template to PCR-amplify variable regions V3 and V4 of the bacterial 16S rRNA gene. The proportion of phyla were different taking into account HPV status (p< 0.0001). In HPV- cervices, the predominant phyla were *Firmicutes* (74.7%), followed by *Actinobacteria* (12.7%), *Bacterioidetes* (5.1%) and *Fusobacteria* (5.1%). On the other hand, in HPV+ samples the same phyla were present, but at different percentages: *Firmicutes* (68.3%), *Actinobacteria* (27.3%), *Fusobacteria* (2.5%) and *Bacterioidetes* (0.7%) (**Figure 1A**). The proportion of phyla was also different *(p*< 0.0001) when comparing AV- (*Firmicutes* (65.8%), *Actinobateria* (27.2%) and *Fusobacterium* (3%)) and AV+ samples *(Firmicutes* (85%), *Fusobacterium* (6%) and *Bacterioidetes* (5.7%)) (**Figure 1B**).

**Figure 2:**
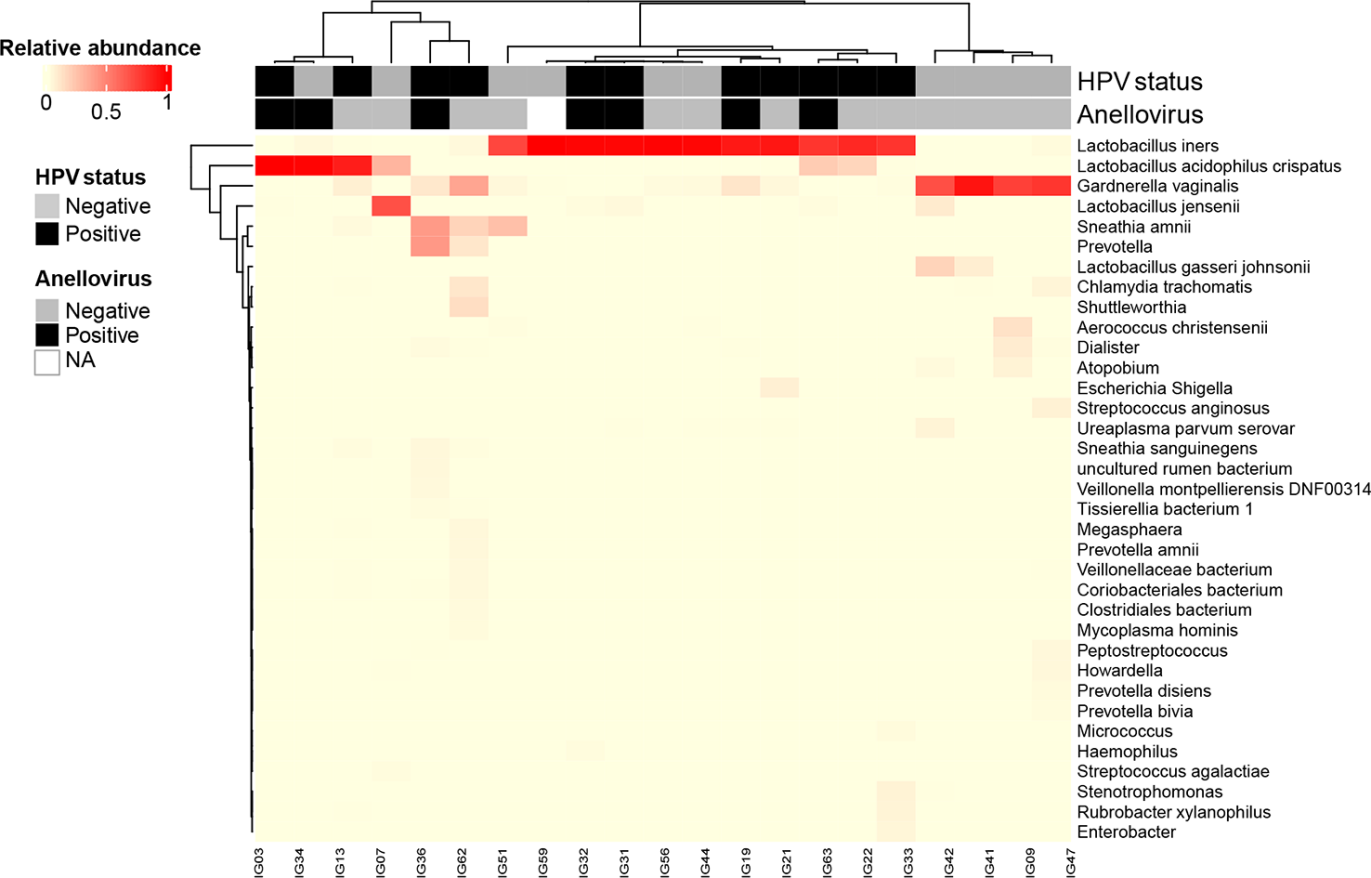
Heatmap generated by unsupervised hierarchical clustering analysis of cervical bacteriomes including HPV and Anellovirus status. CSTs were defined using clustering based on Bray-Curtis dissimilarity and average linkage and then grouped based on vaginal health state in: healthy state – *L.* no-iners dominated (grouping CSTs I and V); transitional state – *L. iners* dominated (CST III); and dysbiosis state – No-Lacto dominated (CST IV). Relative bacterial abundance, HPV and Anellovirus statuses are color-coded according to the legend at the left of the Figure. All taxa shown in the graph presented relative abundance >1%. NA: not available. .

**Figure 3:**
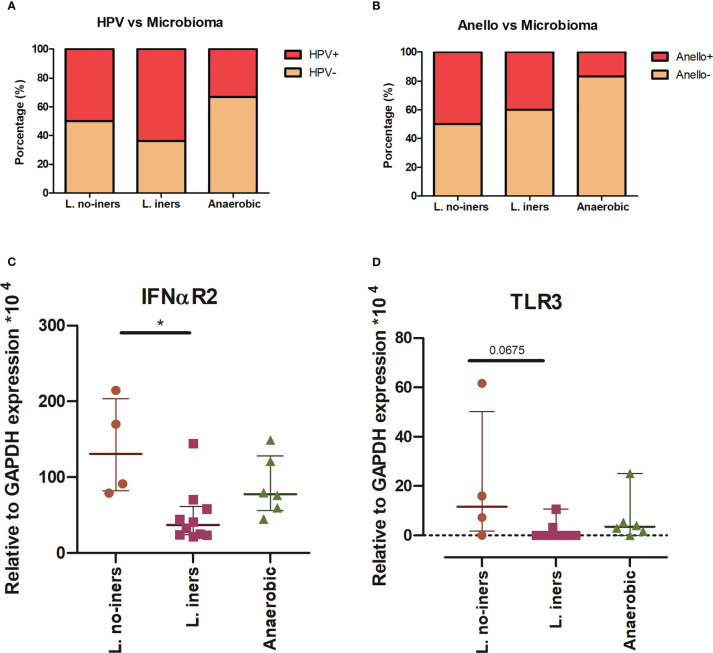
CST distribution related to virus status **(A, B)** and gene expression **(C, D)**. Stacked bar graph of grouped CSTs showing the percentage of HPV **(A)** and Annelovirus **(B)** infected and non-infected patients. Statistical Test: qui-square. Scatter plot of IFNαR2 **(C)** and TLR3 **(D)** expression in *L.* no-iners, *L. iners* and No-Lacto groups. Central horizontal bars represent the median and upper and lower horizontal bars the interquartile range. We show only genes for which we rejected null hypothesis (Kruskal-Wallis test, **(C)** p = 0.0019 and **(D)** p = 0.0291). We performed Dunn’s multiple comparisons as a *post-hoc* test (* - *p* < 0.05).

We documented the presence of four distinct CSTs based on bacteriome hierarchical cluster analysis: CST I (*L. crispatus* dominant, n = 3; 14.2%), CST III (*L. iners* dominant, n = 11; 52.4%), CST IV (anaerobic dominant: *Garnerella*, n = 5; 23.8% and *Sneathia*, n = 1; 4.8%) and CST V (*L. jensenii* dominant, n = 1; 4.8%) (**Figure 2**). Since our sample size is small, we clustered the women in three groups: *L.* no-iners (grouping CST I and V), *L. iners* (CST III) and anaerobic (CST IV). Women within the *L. iners* group reported the use of condoms more frequently and had their first sexual intercourse earlier than women in the anaerobic group (*p* = 0.049). On the other hand, women within the anaerobic group smoke/smoked more than *L. iners* group (*p* = 0.026). In this sense, smoking increased the chance of anaerobic cervical dominance compared with the *L. iners* group (OR = 22.5, 95% CI = 1.6 – 314.6, *p* = 0.0207) (**Supplementary Table 1**). Moreover, anaerobic-colonized cervices expressed higher levels of TLR3 compared with the *L. iners* group (OR = 13.3, 95% CI = 1.1 – 166.4; p = 0.044) (**Supplementary Table 1**). We did not find association between CSTs and HPV or AV infection (**Figures 3A, B**). However, when we assessed gene expression in CSTs independent of HPV or AV status, cervical cells in a *L.* no-iners dominated mucosa expressed higher levels of IFNαR2 and TLR3 compared to the *L. iners* dominated group (**Figures 3C, D**).

We carried out correlation analysis comparing viral and bacterial abundance and observed strong negative correlation between HPV abundance and the *Sphingomonas* genus (r = -0.733, *p* = 0.039) as well as between AV abundance and *Fusobacterium* genus (r = -0.794, *p* = 0.033), while AV abundance showed a strong positive correlation with the presence of *Shuttleworthia* genus (r = 0.771, *p* = 0.042). We also performed correlations between the 61 bacterial genera found in the samples and the expression of assessed genes. We found 37 moderate correlations involving 22 genera and 9 innate immunity genes (**Table 4**). RLRs (RIG-I and MDA5) and their regulators (TRIM25 and RNF125) were involved in 30 of these correlations, where RIG-I and TRIM25 correlated to 11 genera each, 9 of these were common to both genes and 2 were common to RIG-I, TRIM25 and MDA5 (**Table 4**). Finally, we investigated further correlations among the 22 bacterial genera identified above and detected 54 correlations (**Table 5**). Interestingly, genera that correlated with TLRs, UCHL1 and type I IFN receptor (IFNαR2) had no more than three correlations with other genera. On the other hand, genera that correlated to IFNγ, RLRs and their regulators, correlated with three or more genera and mainly were correlated with each other (**Table 5**). Collectively, these data suggest an association between the mRNA expression of RIG-I, MDA5 and their regulators (TRIM25 and RNF125) with anaerobic bacterial abundance and diversity.

**Table 4:** Correlation analyses between bacterial genera abundance and immune gene expression levels.

Genus	RIG-I	TRIM25	MDA5	IFNαR2	IFNγ	RNF125	TLR4	TLR9	UCHL1
***Aerococcus***	–	–	–	–	–	-.563^*^	–	–	–
***Bulleidia***	-	.451^*^	-	-	-	-	-	-	-
***Campylobacter***	.467^*^	–	.465^*^	–	–	–	–	–	–
***Corynebacterium***	-	-	-	-	-	.661^*^	-	-.496^*^	-
**DNF00809**	.570^**^	.572^**^	–	–	–	–	–	–	–
***Enterobacter***	-	-	-	-.479^*^	-	-	-	-	-
***Ezakiella***	–	–	.585^**^	–	–	–	–	–	–
***Fusobacterium***	.467^*^	.441^*^	.515^*^	-	-	-	-	-	-
***Howardella***	.479^*^	–	–	–	–	–	–	–	–
***Hydrogenophilus***	-	-	-	-	-	-	-	-.603^**^	-
***Mobiluncus***	–	–	.456^*^	–	–	–	–	–	–
***Mycoplasma***	.561^**^	.566^**^	-	-	-	-	-	-	-
***Paenibacillus***	-.455^*^	-.480^*^	–	–	–	–	–	–	–
***Parvimonas***	.581^**^	.502^*^	-	-	-	-	-	-	-
***Peptoniphilus***	–	–	–	–	.503^*^	–	–	–	–
***Prevotella***	.561^**^	.600^**^	-	-	.480^*^	-	-	-	-
***Porphyromonas***	.470^*^	.480^*^	.567^**^	–	–	–	–	–	–
***Rubrobacter***	-	-	-	-	-	-	-		.577^*^
***Shuttleworthia***	–	.446^*^	–	–	–	–	–	–	–
***Sneathia***	.464^*^	.645^**^	-	-	-	-	-	-	-
***Staphylococcus***	–	–	–	–	–	.547^*^	–	–	–
***Stenotrophomonas***	-.607^**^	-.526^*^	-	-	-	-	-.556^**^	-	-

r = correlation coefficient; -: comparison with non-significative correlation; Spearman Correlation Degree: strong correlation: 0.7 ≤ r ≤1 or -1 ≤ r ≤ -0.7; moderate correlation: 0.4 ≤ r < 0.7 or -0.7 ≤ r < -0.4; weak correlation: 0 < r < 0.4 or 0.4 < r <0; negative correlation: r < 0. * p < 0.05; ** p < 0.01.

**Table 5:** Correlation analysis between bacterial genera abundance.

GENERA	*Aerococcus*	*Bulleidia*	*Campylobacter*	*Corynebacterium*	DNF00809	*Enterobacter*	*Ezakiella*	*Fusobacterium*	*Howardella*	*Hydrogenophilus*	*Mobiluncus*	*Mycoplasma*	*Paenibacillus*	*Parvimonas*	*Peptoniphilus*	*Prevotella*	*Porphyromonas*	*Shuttleworthia*	*Sneathia*	*Staphylococcus*	*Stenotrophomonas*
***Aerococcus***		**-**	**-**	**-**	**-**	**-**	**-**	**-**	**-**	**-**	**-**	**-**	**-**	**-**	**-**	**-**	**-**	**-**	.645^**^	-.554^**^	**-**
***Bulleidia***			.532^*^	**-**	.740^**^	**-**	**-**	.639^**^	.587^**^	**-**	**-**	.743^**^	**-**	.682^**^	**-**	.531^*^	.530^*^	**-**	.554^**^	**-**	**-**
***Campylobacter***				**-**	**-**	**-**	.462^*^	**-**	.885^**^	**-**	.604^**^	**-**	**-**	.526^*^	.649^**^	.738^**^	.932^**^	**-**	**-**	**-**	**-**
***Corynebacterium***					**-**	**-**	.568^**^	.727^**^	**-**	**-**	.537^*^	**-**	**-**	**-**	**-**	**-**	**-**	**-**	**-**	**-**	**-**
**DNF00809**						**-**	**-**	**-**	**-**	**-**	**-**	.997^**^	**-**	.892^**^	**-**	.602^**^	**-**	**-**	.520^*^	**-**	**-**
***Enterobacter***							**-**	**-**	**-**	**-**	**-**	**-**	**-**	**-**	**-**	**-**	.776^**^	**-**	**-**	.550^**^	**-**
***Ezakiella***								**-**	**-**	**-**	.837^**^	**-**	**-**	**-**	.440^*^	**-**	.626^**^	**-**	**-**	.455^*^	**-**
***Fusobacterium***									**-**	.841^**^	**-**	**-**	**-**	.539^*^	**-**	.649^**^	**-**	**-**	**-**	**-**	**-**
***Howardella***										**-**	**-**	**-**	**-**	.602^**^	.535^*^	.655^**^	.795^**^	**-**	**-**	**-**	**-**
***Hydrogenophilus***											**-**	**-**	.614^**^	**-**	**-**	**-**	**-**	**-**	**-**	**-**	**-**
***Mobiluncus***												**-**	**-**	**-**	.477^*^	.604^**^	.569^**^	**-**	**-**	**-**	**-**
***Mycoplasma***													**-**	.895^**^	**-**	**-**	**-**	**-**	.525^*^	**-**	**-**
***Paenibacillus***														**-**	**-**	**-**	**-**	**-**	**-**	**-**	**-**
***Parvimonas***															.478^*^	.711^**^	.476^*^	.435^*^	.567^**^	**-**	**-**
***Peptoniphilus***																.748^**^	.650^**^	**-**	**-**	**-**	**-**
***Prevotella***																	.756^**^	**-**	.534^*^	**-**	**-**
***Porphyromonas***																		**-**	**-**	**-**	-.433^*^
***Shuttleworthia***																			**-**	**-**	**-**
***Sneathia***																				**-**	**-**
***Staphylococcus***																					**-**
***Stenotrophomonas***																					

r = correlation coefficient; -: comparison with non-significative correlation; only correlations between genera that correlated to gene expression levels (**Table 4**) are shown; Spearman Correlation Degree: strong correlation: 0.7 ≤ r ≤1 or -1 ≤ r ≤ -0.7; moderate correlation: 0.4 ≤ r < 0.7 or -0.7 ≤ r < -0.4; weak correlation: 0 < r < 0.4 or 0.4 < r <0; negative correlation: r < 0. * p < 0.05; ** p < 0.01. Rubrobacter was not presented here as no correlations were found between this genus and other genera.

## 4 Discussion

Cervical cancer development is associated with HPV infection, which progression depends on the cervical milieu. Herein, we tried to elucidate part of the interplay between host innate immunity and the microbiome (virome and bacteriome) using a Brazilian convenience sample. First, we assessed whether social and immune factors could impact HPV infection and found that IFN-I expression correlated to that of different innate immunity genes depending on HPV status. Virome data from cervical samples revealed two viral families: *Papillomaviridae* and *Anelloviridae* and allowed the assembly of seven complete HPV genome sequences. The most prevalent HPV was HPV16 (6/11 women). Some samples were also infected by multiple HPV types. Bacteriome analysis identified 12 phyla that were differently distributed depending on HPV or AV presence. Overall, behavioral factors and innate immune gene expression were associated to *L. iners* and anaerobic dominated mucosa. Expression of RLRs and their regulators correlated to several genera abundance which in turn, correlated to each other, suggesting that this receptor family triggers innate immune signaling that may impact vaginosis.

Known risk factors for cervical cancer development (41, 42) were confirmed in our samples where the occurrence of previous STI increased eight times the risk for HPV infection. Higher expression of IFNα and IFNβ and lower levels of MDA5 and TLR3 were linked to HPV infection. HPV infection changes expression of several genes linked to innate immune response (43–46) and our correlation analysis suggest that in the presence of HPV, the relationship between these genes is altered. Changes in TLR’s and RLR’s mRNA and protein levels have been assessed in other HPV-associated cancer models. In HPV-associated oropharyngeal squamous cell carcinoma (OPSCC), TLR4 protein expression was lower while higher levels of TLR9 mRNA were observed (47). In our study, higher expression of TLR9 mRNA was found in the HPV+ cervical samples, but no changes in TLR4 expression were seen (12). Overall, in the absence of HPV a negative correlation between TRL4 and TLR9 expression was seen, while a positive one occurred in HPV+ samples. Taken together, the correlation analyses between HPV+ and HPV- women reinforces the idea that regulation of IFNs and PRRs expression during HPV infection could play a pivotal role in inflammatory process, creating a favorable environment for HPV persistence, cancer development and HIV susceptibility, which has been described in the literature by us and others (12, 48–51). The abundance of HPV was negatively correlated to UCHL1’s expression contradicting Karim et al. (2013) (11), who used a primary keratinocyte HPV16 infection model and concluded that HPV dampens host innate immunity *via* UCHL1 induction.

In this study, the number of sexual partners and number of HPV reads were correlated to AV reads, reinforcing idea that these viruses are sexually transmitted (52–54). There was a higher prevalence of AVs in HPV+ women and literature associate these coinfections not only in cervix, but also at other sites (53–55). It has been suggested that coinfection contributes to laryngeal carcinoma progression (55, 56) and magnifies immune responses (57–59), creating a microenvironment that favours their establishment and facilitates tumor development.

Upon bacteriome determination, the studied cervical samples were classified into three groups: *L.* no-iners (healthy); *L. iners* (transitional) and anaerobic (dysbiosis). In our samples, smoking was the only behavioral factor linked to CST IV (anaerobic), but a larger sample size is needed to perform a more robust analysis. The association between cervical microbial diversity (CST III and IV) and HPV infection/cervical dysplasia has been described (3, 25, 26); however, we did not find this association. Independently of HPV, the cervices assessed here were dominated by the same bacterial phyla, although at different proportions in HPV-infected and uninfected women. Similarly, a Chinese study that analyzed 276 cervical smear samples found that age and HPV infection were associated with microbiota structure and diversity (60). When we compared AV+ and AV- samples, bacterial proportion and phyla were different between the two groups. The great dominance of *Firmicutes* and small proportion of other phyla indicates the dominance of *Lactobacillus* spp. and low diversity in AV+ group, opposite to what was observed in the literature, where AV abundance was associated to non-*Lactobacillus* (59). Our data also indicates that a *L.* no-iners dominated mucosa expressed higher TLR3 and IFNαR2 than *L. iners* mucosa. It is known that different mucosa are colonized by microorganisms that are in close contact with epithelial cells. In human colon for instance, TLR3 is concentrated in mature columnar cells that create a luminal barrier protection surface (61). LPS and Gram-positive bacteria promote upregulation of TLR3 (61, 62) and IFNβ expressions when intestinal cell lines are exposed simultaneously to *B. bifidum* and poly(I:C) (61). Interestingly, TLR3 seems to be able to discriminate between commensal and pathogenic bacteria in mice gastrointestinal mucosa, where it is associated with an anti-inflammatory response after recognition of commensal bacterial DNA (61, 63). Little is known about the role of TLR3 in cervix, however it is possible that TLR3 acts in the maintenance of an anti-inflammatory environment in *L.* no-iners dominated mucosa. Concerning the expression of IFNαR2 and its association with *L.* no-iners mucosa, no information is available in the literature. It is possible that this receptor acts synergistically with TLR3 in maintaining a healthy mucosa, but this needs to be further evaluated.

Still in the bacteriome context, we observed that abundance of certain bacteria genera found in CST IV correlated with others in the same CST and some genera positively correlated to innate immunity genes, mainly genes involved in RLR-mediated pathway. We were surprised with the observations that RLRs and not TLRs expressions were altered in our samples (64, 65). RLRs are cytoplasmic PAMP receptors initially characterized as viral RNA sensors (*e.g.*, short and long dsRNA, 5′-triphosphorylated RNA, ssRNA and poly(I:C)) and their role in DNA and bacterial recognition is emerging (64, 65). For instance, *Salmonella typhimurium* and *Listeria monocytogenes* nucleic acids can activate a RIG-I-mediated response in non-phagocytic and phagocytic cells (66–69). Human microglial cells also have RIG-I upregulated at protein levels when infected with either *N*. *meningitidis*, *S*. *aureus*, or *S. pneumoniae*. The same effect was obtained when DNA from these bacteria was used as a PAMP, and the end result was induction of type I IFN (70). More recently, the role of RIG-I in LPS-induced innate immune activation has been shown in endothelial cells. Surprisingly, this activation was independent of TLR4 LPS detection and was also independent of RIG-I RNA detection (71). The same group showed that different endothelial cells can respond to LPS in either a TLR4- or a RIG-I-dependent manner which resulted in different adhesion molecules expression (72). In mice, RIG-I depletion caused an alteration in the gut bacteriome and decreased gut IgA secretion. The latter is crucial for maintaining immunological homeostasis between intestinal bacteria and mucosa (18). We find intriguing that expression of either RIG-I and MDA5 or its regulators TRIM25 and RNF125 were correlated to some bacterial genera abundance, linking the importance of these PRRs in mucosal homeostasis and wonder if these proteins exert their function in a similar way in the gut and in the cervix. In this context, it will be interesting to analyse the mucosa in RIG-I-deficient female mice.

Taken together, despite the lack of adjustments for multiple comparisons and lack of control for covariates due to limited sample size, our data suggest differences in bacterial composition depending on HPV or AV status. Moreover, correlations between bacterial genera and immune gene expression were found, linking RLRs function to dysbiosis. Longitudinal studies and a larger sample size would further confirm the results presented here and would allow to determine the causal relationship between bacteria abundance and innate immunity.

## Data availability statement

The datasets presented in this study can be found in online repositories. The names of the repository/repositories and accession number(s) can be found below: PRJNA933917 (SRA).

## Ethics statement

The studies involving human participants were reviewed and approved by Ethical Committee of Instituto de Puericultura e Pediatria Martagão Gesteira, UFRJ (approval number: 49035215.4.0000.5264). The patients/participants provided their written informed consent to participate in this study.

## Author contributions

AB, AG, EM and MS: conceptualization and writing original draft. AB, CP, AM, YF, and GA: sample collection and processing. AB, CP, AG, and EM: gene expression experiment and analysis. AB, LG, JS, GC: virome and bacteriome experiment and analysis. MS, AG, and EM: funding acquisition. AB, AG, EM, LG, JS, GC and MS: writing, reviewing, and editing. All authors contributed to the article and approved the submitted version.

## References

[1] INCA. Estimativa/2020 - incidência de câncer no Brasil. 1st ed. Rio de Janeiro: Ministério da Saúde (2019) p. 1–120 p.

[2] Bruni L Albero G Serrano B Mena M Collado J Gómez D. Human papillomavirus and related diseases report world. Barcelona: ICO/IARC Inf Centre HPV Cancer (HPV Inf Centre) (2021), 1–303. Available at: www.hpvcentre.net.

[3] Mitra A MacIntyre DA Marchesi JR Lee YS Bennett PR Kyrgiou M. The vaginal microbiota, human papillomavirus infection and cervical intraepithelial neoplasia: what do we know and where are we going next? Microbiome (2016) 4(1):58. doi: 10.1186/S40168-016-0203-0 27802830 PMC5088670

[4] Walboomers JM Jacobs M v Manos MM Bosch FX Kummer JA Shah K v. Human papillomavirus is a necessary cause of invasive cervical cancer worldwide. J Pathol (1999) 189(1):12–9. doi: 10.1002/(SICI)1096-9896(199909)189:1<12::AID-PATH431>3.0.CO;2-F 10451482

[5] Lin D Kouzy R Jaoude JA Noticewala SS Delgado Medrano AY Klopp AH. Microbiome factors in HPV-driven carcinogenesis and cancers. PloS Pathog (2020) 16(6):e1008524. doi: 10.1371/JOURNAL.PPAT.1008524 32497113 PMC7271998

[6] Nguyen P v Kafka JK Ferreira VH Roth K Kaushic C. Innate and adaptive immune responses in male and female reproductive tracts in homeostasis and following HIV infection. Cell Mol Immunol (2014) 11(5):410–27. doi: 10.1038/cmi.2014.41 PMC4197208 24976268

[7] Wicherska-pawłowska K Wróbel T Rybka J. Toll-like receptors (TLRs), NOD-like receptors (NLRs), and RIG-I-Like receptors (RLRs) in innate immunity. TLRs, NLRs, and RLRs ligands as immunotherapeutic agents for hematopoietic diseases. Int J Mol Sci (2021) 22(24):13397. doi: 10.3390/IJMS222413397 34948194 PMC8704656

[8] Gack MU Shin YC Joo Ch Urano T Liang C Sun L. TRIM25 RING-finger E3 ubiquitin ligase is essential for RIG-i-mediated antiviral activity. Nature (2007) 446(7138):916–20. doi: 10.1038/nature05732 17392790

[9] Arimoto Ki Takahashi H Hishiki T Konishi H Fujita T Shimotohno K. Negative regulation of the RIG-I signaling by the ubiquitin ligase RNF125. PNAS (2007) 104(18):7500–5. doi: 10.1073/pnas.0611551104 PMC1863485 17460044

[10] Liao Z Su J. Progresses on three pattern recognition receptor families (TLRs, RLRs and NLRs) in teleost. Dev Comp Immunol (2021) :122:104131. doi: 10.1016/J.DCI.2021.104131 34022258

[11] Karim R Tummers B Meyers C Biryukov JL Alam S Backendorf C. Human papillomavirus (HPV) upregulates the cellular deubiquitinase UCHL1 to suppress the keratinocyte’s innate immune response. PloS Pathog (2013) 9(5):e1003384. doi: 10.1371/journal.ppat.1003384 23717208 PMC3662672

[12] Britto AMA Goes LR Sivro A Policarpo C Meirelles ÂR Furtado Y. HPV induces changes in innate immune and adhesion molecule markers in cervical mucosa with potential impact on HIV infection. Front Immunol (2020) 11:2078/full. doi: 10.3389/fimmu.2020.02078/full 33013878 PMC7494736

[13] Silva J Cerqueira F Medeiros R. Chlamydia trachomatis infection: implications for HPV status and cervical cancer. Arch Gynecol Obstet (2014) 289(4):715–23. doi: 10.1007/s00404-013-3122-3 24346121

[14] Uysal IB Boué V Murall CL Graf C Selinger C Hirtz C. Concomitant and productive genital infections by HSV-2 and HPV in two young women: a case report. IDCases (2022) 30:e01604. doi: 10.1016/j.idcr.2022.e01604 36119756 PMC9478384

[15] France M Alizadeh M Brown S Ma B Ravel J. Towards a deeper understanding of the vaginal microbiota. Nat Microbiol (2022) 7(3):367. doi: 10.1038/s41564-022-01083-2 35246662 PMC8910585

[16] Rezasoltani S Ghanbari R Looha MA Mojarad EN Yadegar A Stewart D. Expression of main toll-like receptors in patients with different types of colorectal polyps and their relationship with gut microbiota. Int J Mol Sci (2020) 21(23):8968. doi: 10.3390/ijms21238968 33255933 PMC7729598

[17] Fischer JC Bscheider M Eisenkolb G Lin CC Wintges A Otten V. RIG-I/MAVS and STING signaling promote gut integrity during irradiation- and immune-mediated tissue injury. Sci Transl Med (2017) 9(386):eaag2513. doi: 10.1126/scitranslmed.aag2513 28424327 PMC5604790

[18] Zhu H Xu WY Hu Z Zhang H Shen Y Lu S. RNA Virus receptor rig-I monitors gut microbiota and inhibits colitis-associated colorectal cancer. J Exp Clin Cancer Res (2017) 36(1):1–11. doi: 10.1186/s13046-016-0471-3 28057020 PMC5217425

[19] Ravel J Gajer P Abdo Z Schneider GM Koenig SSK McCulle SL. Vaginal microbiome of reproductive-age women. Proc Natl Acad Sci U.S.A. (2011) 108(SUPPL. 1):4680–7. doi: 10.1073/pnas.1002611107 PMC3063603 20534435

[20] Petrova MI Lievens E Malik S Imholz N Lebeer S. Lactobacillus species as biomarkers and agents that can promote various aspects of vaginal health. Front Physiol Front Media S.A (2015) 6:81. doi: 10.3389/fphys.2015.00081 PMC4373506 25859220

[21] Hellberg D Nilsson S Mårdh PA. Bacterial vaginosis and smoking. Int J STD AIDS. (2000) 11(9):603–6. doi: 10.1258/0956462001916461 10997505

[22] Sivro A Mwatelah R Kambaran C Gebrebrhan H Becker MG Ma H. Sex work is associated with increased vaginal microbiome diversity in young women from Mombasa, Kenya. J Acquir Immune Defic Syndr (2020) 85(1):79–87. doi: 10.1097/QAI.0000000000002406 32433252 PMC12506801

[23] Fethers KA Fairley CK Hocking JS Gurrin LC Bradshaw CS. Sexual risk factors and bacterial vaginosis: a systematic review and meta-analysis. Clin Infect Dis (2008) 47(11):1426–35. doi: 10.1086/592974 18947329

[24] Borgdorff H van der Veer C Van Houdt R Alberts CJ De Vries HJ Bruisten SM. The association between ethnicity and vaginal microbiota composition in Amsterdam, the Netherlands. PloS One (2017) 12(7):e0181135. doi: 10.1371/journal.pone.0181135 28700747 PMC5507447

[25] Curty G de Carvalho PS Soares MA. The role of the cervicovaginal microbiome on the genesis and as a biomarker of premalignant cervical intraepithelial neoplasia and invasive cervical cancer. Int J Mol Sci (2019) 21(1):222. doi: 10.3390/ijms21010222 31905652 PMC6981542

[26] Mitra A MacIntyre DA Lee YS Smith A Marchesi JR Lehne B. Cervical intraepithelial neoplasia disease progression is associated with increased vaginal microbiome diversity. Sci Rep (2015) 5(1):16865. doi: 10.1038/srep16865 26574055 PMC4648063

[27] Petrova MI van den Broek M Balzarini J Vanderleyden J Lebeer S. Vaginal microbiota and its role in HIV transmission and infection. FEMS Microbiol Rev (2013) 37(5):762–92. Available at: https://academic.oup.com/femsre/article/37/5/762/542986. 10.1111/1574-6976.12029 23789590

[28] Marconi C El-Zein M Ravel J Ma B Lima MD Carvalho NS. Characterization of the vaginal microbiome in women of reproductive age from 5 regions in Brazil. Sex Transm Dis (2020) 47(8):562–9. doi: 10.1097/OLQ.0000000000001204 32520883

[29] Jakobsen RR Haahr T Humaidan P Jensen JS Kot WP Castro-Mejia JL. Characterization of the vaginal DNA virome in health and dysbiosis. Viruses (2020) 12:1143. doi: 10.3390/v12101143 33050261 PMC7600586

[30] Happel AU Varsani A Balle C Passmore JA Jaspan H. The vaginal virome-balancing female genital tract bacteriome, mucosal immunity, and sexual and reproductive health outcomes? Viruses (2020) 12(8):832. doi: 10.3390/v12080832 32751611 PMC7472209

[31] Siqueira J Curty G Xutao D Hofer C Machado E Seuánez H. Composite analysis of the virome and bacteriome of HIV/HPV Co-infected women reveals proxies for immunodeficiency. Viruses (2019) 11(5):422. doi: 10.3390/v11050422 31067713 PMC6563245

[32] Britto AMA Policarpo C Pezzuto P Meirelles ARI Furtado YL Almeida G. Detection of sexually transmitted infections at a Brazilian gynecology center: high prevalence of co-infections. J Bras Patol Med Lab (2018) 54(6):393–400. doi: 10.5935/1676-2444.20180060

[33] Joshi N Fass J. Sickle: a sliding-window, adaptive, quality-based trimming tool for FastQ files (Version 1.33) [Software] (2011). Available at: https://github.com/najoshi/sickle.

[34] Li H Durbin R. Fast and accurate short read alignment with burrows–wheeler transform. Bioinformatics (2009) 25(14):1754. doi: 10.1093/bioinformatics/btp324 19451168 PMC2705234

[35] Camacho C Coulouris G Avagyan V Ma N Papadopoulos J Bealer K. BLAST+: architecture and applications. BMC Bioinf (2009) 10:421. doi: 10.1186/1471-2105-10-421 PMC2803857 20003500

[36] Callahan BJ McMurdie PJ Rosen MJ Han AW Johnson AJA Holmes SP. DADA2: high resolution sample inference from illumina amplicon data. Nat Methods (2016) 13(7):581. doi: 10.1038/nmeth.3869 27214047 PMC4927377

[37] Bolyen E Rideout JR Dillon MR Bokulich NA Abnet CC Al-Ghalith GA. Reproducible, interactive, scalable and extensible microbiome data science using QIIME 2. Nat Biotechnol (2019) 37(8):852. doi: 10.1038/s41587-019-0209-9 31341288 PMC7015180

[38] McDonald D Price MN Goodrich J Nawrocki EP Desantis TZ Probst A. An improved greengenes taxonomy with explicit ranks for ecological and evolutionary analyses of bacteria and archaea. ISME J (2012) 6(3):610–8:3. doi: 10.1038/ismej.2011.139 PMC3280142 22134646

[39] Schober P Boer C Schwarte LA. Correlation coefficients: appropriate use and interpretation. Anesth Analg (2018) 126(5):1763–8. doi: 10.1213/ANE.0000000000002864 29481436

[40] Castro F Cardoso AP Gonçalves RM Serre K Oliveira MJ. Interferon-gamma at the crossroads of tumor immune surveillance or evasion. Front Immunol (2018) 1. doi: 10.3389/fimmu.2018.00847 PMC5945880 29780381

[41] Zhang S Xu H Zhang L Qiao Y. Cervical cancer: epidemiology, risk factors and screening. Chin J Cancer Res (2020) 32(6):720. doi: 10.21147/j.issn.1000-9604.2020.06.05 33446995 PMC7797226

[42] Mekuria M Edosa K Endashaw M Bala ET Chaka EE Deriba BS. Prevalence of cervical cancer and associated factors among women attended cervical cancer screening center at gahandi memorial hospital, Ethiopia. Cancer Inform (2021) 20:1–6. doi: 10.1177/11769351211068431 PMC8725021 34992337

[43] de Matos LGG Cândido EB Vidigal PVT Bordoni PHC Lamaita RM Carneiro MM. Association between toll-like receptor and tumor necrosis factor immunological pathways in uterine cervical neoplasms. Tumori J (2017) 103(1):81–6. doi: 10.5301/tj.5000576 28009429

[44] Hasimu A Ge L Li QZ Zhang RP Guo X. Expressions of toll-like receptors 3, 4, 7, and 9 in cervical lesions and their correlation with HPV16 infection in uighur women. Chin J Cancer (2011) 30(5):344–50. doi: 10.5732/cjc.010.10456 PMC4013399 21527067

[45] Chiang C Pauli EK Biryukov J Feister KF Meng M White EA. The human papillomavirus E6 oncoprotein targets USP15 and TRIM25 to suppress RIG-I-Mediated innate immune signaling. J Virol (2018) 92(6):e01737–17. doi: 10.1128/JVI.01737-17 PMC5827370 29263274

[46] DeCarlo CA Rosa B Jackson R Niccoli S Escott NG Zehbe I. Toll-like receptor transcriptome in the HPV-positive cervical cancer microenvironment. Clin Dev Immunol (2012) 2012:785825. doi: 10.1155/2012/785825 22013487 PMC3195758

[47] Tobouti PL Bolt R Radhakrishnan R Sousa SCOM Hunter KD Tobouti PL. Altered toll-like receptor expression and function in HPV-associated oropharyngeal carcinoma. Oncotarget (2018) 9(1):236–48. doi: 10.18632/oncotarget.18959 PMC5787461 29416610

[48] Cannella F Pierangeli A Scagnolari C Cacciotti G Tranquilli G Stentella P. TLR9 is expressed in human papillomavirus-positive cervical cells and is overexpressed in persistent infections. Immunobiology (2014) S0171-2985(14):00203–4. doi: 10.1016/j.imbio.2014.10.012 25454809

[49] Ferreira AR Ramalho AC Marques M Ribeiro D. The interplay between antiviral signalling and carcinogenesis in human papillomavirus infections. Cancers (Basel) (2020) 12(3):646. doi: 10.3390/cancers12030646 32164347 PMC7139948

[50] Li J Rao H Jin C Liu J. Involvement of the toll-like Receptor/Nitric oxide signaling pathway in the pathogenesis of cervical cancer caused by high-risk human papillomavirus infection. BioMed Res Int (2017) 2017:1–8. doi: 10.1155/2017/7830262 PMC5463171 28626766

[51] Liebenberg LJP McKinnon LR Yende-Zuma N Garrett N Baxter C Kharsany ABM. HPV infection and the genital cytokine milieu in women at high risk of HIV acquisition. Nat Commun (2019) 10(1):5227. doi: 10.1038/s41467-019-13089-2 31745084 PMC6863918

[52] Fornai C Maggi F Vatteroni ML Pistello M Bendinelli M. High prevalence of TT virus (TTV) and TTV-like minivirus in cervical swabs. J Clin Microbiol (2001) 39(5):2022. doi: 10.1128/JCM.39.5.2022-2024.2001 11326040 PMC88075

[53] Changani L Bouzari M Talebi A. Torque teno mini virus infection in chronic cervicitis and cervical tumors in isfahan, Iran. Intervirology (2013) 56(4):265–70. doi: 10.1159/000348514 23689906

[54] Siahpoush M Noorbazargan H Kalantari S Shayestehpour M Yazdani S. Coinfection of torque teno virus (TTV) and human papillomavirus (HPV) in cervical samples of women living in Tehran, Iran. Iran J Microbiol (2022) 14(2):181. doi: 10.18502/IJM.V14I2.9185 35765558 PMC9168241

[55] Szládek G Juhász A Kardos K Szöke K Major T Sziklai I. High co-prevalence of genogroup 1 TT virus and human papillomavirus is associated with poor clinical outcome of laryngeal carcinoma. J Clin Pathol (2005) 58(4):402. doi: 10.1136/JCP.2004.022103 15790705 PMC1770630

[56] Fehér E Gáll T Murvai M Kis A Boda R Sápy T. Investigation of the occurrence of torque tenovirus in malignant and potentially malignant disorders associated with human papillomavirus. J Med Virol (2009) 81(11):1975–81. doi: 10.1002/jmv.21627 19774682

[57] Rocchi J Ricci V Albani M Lanini L Andreoli E Macera L. Torquetenovirus DNA drives proinflammatory cytokines production and secretion by immune cells *Via* toll-like receptor 9. Virology (2009) 394(2):235–42. doi: 10.1016/j.virol.2009.08.036 19765789

[58] Singh P Ramamoorthy S. Lack of strong anti-viral immune gene stimulation in torque teno sus Virus1 infected macrophage cells. Virology (2016) 495:63–70. doi: 10.1016/j.virol.2016.04.028 27179346 PMC4912913

[59] Kaelin EA Skidmore PT Łaniewski P Holland LA Chase DM Herbst-Kralovetz MM. Cervicovaginal DNA virome alterations are associated with genital inflammation and microbiota composition. mSystems (2022) 7(2):e0006422. doi: 10.1128/msystems.00064-22 35343798 PMC9040584

[60] Hu J Wu Y Quan L Yang W Lang J Tian G. Research of cervical microbiota alterations with human papillomavirus infection status and women age in sanmenxia area of China. Front Microbiol (2022) 13. doi: 10.3389/fmicb.2022.1004664 PMC9608786 36312946

[61] Furrie E Macfarlane S Thomson G Macfarlane GT. Toll-like receptors-2, -3 and -4 expression patterns on human colon and their regulation by mucosal-associated bacteria. Immunology (2005) 115(4):565. doi: 10.1111/j.1365-2567.2005.02200.x 16011525 PMC1782176

[62] Nejsum LN Piec A Fijak M Ernstsen C v. Fischer D Maier TJ. Systemic LPS induces toll-like receptor 3 (TLR3) expression and apoptosis in testicular mouse tissue. Cell Tissue Res (2019) 378(1):143–54. doi: 10.1007/s00441-019-03022-w 30989399

[63] Kawashima T Kosaka A Yan H Guo Z Uchiyama R Fukui R. Double-stranded RNA of intestinal commensal but not pathogenic bacteria triggers production of protective interferon-β. Immunity (2013) 38(6):1187–97. doi: 10.1016/j.immuni.2013.02.024 23791646

[64] Liwinski T Zheng D Elinav E. The microbiome and cytosolic innate immune receptors. Immunol Rev (2020) 297(1):207–24. doi: 10.1111/imr.12901 32658330

[65] Pandey S Kawai T Akira S. Microbial sensing by toll-like receptors and intracellular nucleic acid sensors. Cold Spring Harb Perspect Biol (2015) 7(1):a016246. doi: 10.1101/cshperspect.a016246 PMC4292165 25301932

[66] Schmolke M Patel JR de Castro E Sánchez MTA Uccellini MB Miller JC. RIG-I detects mRNA of intracellular salmonella enterica serovar typhimurium during bacterial infection. mBio (2014) 5(2):e01006–14. doi: 10.1128/MBIO.01006-14 PMC3977358 24692634

[67] Abdullah Z Schlee M Roth S Mraheil MA Barchet W Böttcher J. RIG-I detects infection with live listeria by sensing secreted bacterial nucleic acids. EMBO J (2012) 31(21):4153–64. doi: 10.1038/emboj.2012.274 PMC3492734 23064150

[68] Hagmann CA Herzner AM Abdullah Z Zillinger T Jakobs C Schuberth C. RIG-I detects triphosphorylated RNA of listeria monocytogenes during infection in non-immune cells. PloS One (2013) 8(4):e62872. doi: 10.1371/journal.pone.0062872 23653683 PMC3639904

[69] Imaizumi T Sashinami H Mori F Matsumiya T Yoshida H Nakane A. Listeria monocytogenes induces the expression of retinoic acid-inducible gene-I. Microbiol Immunol (2006) 50(10):811–5. doi: 10.1111/j.1348-0421.2006.tb03857.x 17053317

[70] Johnson MB Halman JR Burmeister AR Currin S Khisamutdinov EF Afonin KA. Retinoic acid inducible gene-I mediated detection of bacterial nucleic acids in human microglial cells. J Neuroinflamm (2020) 17(1):1–14. doi: 10.1186/s12974-020-01817-1 PMC7195775 32357908

[71] Moser J Heeringa P Jongman RM Zwiers PJ Niemarkt AE Yan R. Intracellular RIG-I signaling regulates TLR4-independent endothelial inflammatory responses to endotoxin. J Immunol (2016) 196(11):4681–91. doi: 10.4049/jimmunol.1501819 27183587

[72] Dayang EZ Plantinga J ter Ellen B van Meurs M Molema G Moser J. Identification of LPS-activated endothelial subpopulations with distinct inflammatory phenotypes and regulatory signaling mechanisms. Front Immunol (2019) 10:1169. doi: 10.3389/fimmu.2019.01169 31178871 PMC6543489

