# Supplementary material for: Microbiome analysis of Brazilian women cervix reveals specific bacterial abundance correlation to RIG-like receptor gene expression
Source: Front Immunol. 2023 Apr 25;14:1147950. doi: 10.3389/fimmu.2023.1147950 (PMC10167488; doi:10.3389/fimmu.2023.1147950)
Supplement: Supplementary file 1 [file DataSheet_1.docx]

Supplementary Material

Microbiome analysis of Brazilian women cervix reveals specific bacterial abundance correlation to RIG-like receptor gene expression

Alan Messala A. Britto*, Juliana D. Siqueira, Gislaine Curty, Livia R. Goes, Cintia Policarpo, Angela R. Meyrelles, Yara Furtado, Gutemberg Almeida, Ana Lucia M. Giannini, Elizabeth S. Machado, Marcelo A. Soares

*** Correspondence:** Alan Messala A. Britto: alanmessala@yahoo.com.br

# Supplementary Data


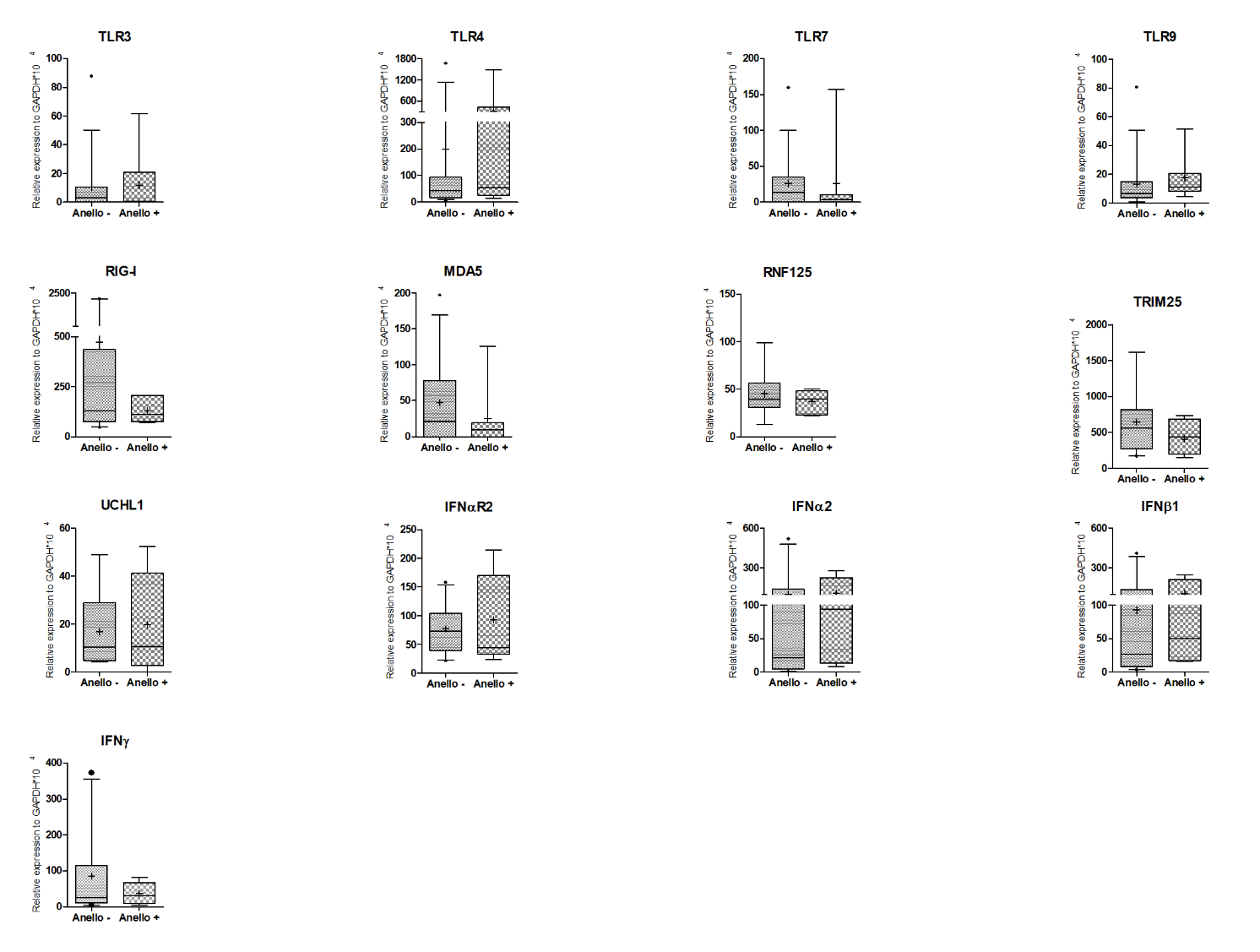


**Supplementary Figure 1:** Innate immunity gene expression levels in Anellovirus-infected and uninfected women. qPCR on cervical samples of Anello- and Anello+ women. Relative expression of target genes was normalized to GAPDH (2−1Ct). Box plots represent 10–90 percentile; dots represent outlier samples; “+” represents the mean and the horizontal bar, the median. No comparison was statistically significant.

# Supplementary Table 1: Odds ratio analysis between sociodemographic and molecular factors and HPV status

|  |  | **HPV status** | |  |  |
| --- | --- | --- | --- | --- | --- |
|  | **Total (22)** | **HPV- (11)** | **HPV+ (11)** | **OR** | **95% CI** |
| **Previous STI (%)** | 9 (40.9) | 2 (22.2) | 7 (77.8) | 7.875 | 1.105 – 56.123 |
| **≥41 years (%)** | 9 (40.9) | 7 (77.8) | 2 (22.2) | 0.127 | 0.018 – 0.905 |
| **High IFNα2 (%)** | 7 (31.8) | 0 (0) | 7 (100) | 38.3 | 1.8 – 820.2 |
| **High IFNβ1 (%)** | 7 (31.8) | 1 (14.3) | 6 (85.7) | 12.0 | 1.1 – 128.8 |
| **High MDA5 (%)** | 6 (27.3) | 6 (100) | 0 (0) | 0.0368 | 0.002 – 0.777 |
| **High TLR3 (%)** | 12 (54.5) | 10 (83.3) | 2 (16.7) | 0.022 | 0.002 – 0.289 |
| **High TLR7 (%)** | 4 (18.2) | 0 (0) | 4 (100) | 0.026 | 0.0012 – 0.558 |
| **Anello+ (%)*** | 7 (33.3) | 1 (14.3) | 6 (85.7) | 10.8 | 1.0 – 117.0 |
|  |  | **Bacteria dominance** | |  |  |
|  | **Total (18)** | ***L. iners* (11)** | **Anaerobic(7)** |  |  |
| **Smoke (%)** | 8 (44.4) | 2 (25) | 6 (75) | 22.5 | 1.6 – 314.6 |
| **High TLR3 (%)** | 9 (50) | 3 (33.3) | 6 (66.7) | 13.3 | 1.1 – 166.4 |

*Virome results including 10 HPV- and 11 HPV+ women
